# Impact of agriculture on the selection of insecticide resistance in the malaria vector *Anopheles gambiae*: a multigenerational study in controlled conditions

**DOI:** 10.1186/s13071-014-0480-z

**Published:** 2014-10-16

**Authors:** Theresia Estomih Nkya, Rodolphe Poupardin, Frederic Laporte, Idir Akhouayri, Franklin Mosha, Stephen Magesa, William Kisinza, Jean-Philippe David

**Affiliations:** Laboratoire d’Ecologie Alpine, UMR CNRS 5553, BP 53, 38041 Grenoble cedex 09, France; Université Grenoble-Alpes, Grenoble, France; National Institute of Medical Research of Tanzania. Amani Medical Research Centre, P. O. Box 81, Muheza, Tanga Tanzania; Department of Vector Biology, Liverpool School of Tropical Medicine, Pembroke place, L35QA Liverpool, UK; KCM College of Tumaini University, P. O. Box. 2240, Moshi, Tanzania; RTI International-Tanzania, P.O.Box 369, Dar es Salaam, Tanzania

**Keywords:** Agriculture, Pesticide, Pollutant, Mosquito, Anopheles gambiae, Malaria, Insecticide resistance, Detoxification enzymes, Cuticle, *Kdr* mutation

## Abstract

**Background:**

Resistance of mosquitoes to insecticides is mainly attributed to their adaptation to vector control interventions. Although pesticides used in agriculture have been frequently mentioned as an additional force driving the selection of resistance, only a few studies were dedicated to validate this hypothesis and characterise the underlying mechanisms. While insecticide resistance is rising dramatically in Africa, deciphering how agriculture affects resistance is crucial for improving resistance management strategies. In this context, the multigenerational effect of agricultural pollutants on the selection of insecticide resistance was examined in *Anopheles gambiae*.

**Methods:**

An urban Tanzanian *An. gambiae* population displaying a low resistance level was used as a parental strain for a selection experiment across 20 generations. At each generation larvae were selected with a mixture containing pesticides and herbicides classically used in agriculture in Africa. The resistance levels of adults to deltamethrin, DDT and bendiocarb were compared between the selected and non-selected strains across the selection process together with the frequency of *kdr* mutations. A microarray approach was used for pinpointing transcription level variations selected by the agricultural pesticide mixture at the adult stage.

**Results:**

A gradual increase of adult resistance to all insecticides was observed across the selection process. The frequency of the L1014S *kdr* mutation rose from 1.6% to 12.5% after 20 generations of selection. Microarray analysis identified 90 transcripts over-transcribed in the selected strain as compared to the parental and the non-selected strains. Genes encoding cuticle proteins, detoxification enzymes, proteins linked to neurotransmitter activity and transcription regulators were mainly affected. RT-qPCR transcription profiling of candidate genes across multiple generations supported their link with insecticide resistance.

**Conclusions:**

This study confirms the potency of agriculture in selecting for insecticide resistance in malaria vectors. We demonstrated that the recurrent exposure of larvae to agricultural pollutants can select for resistance mechanisms to vector control insecticides at the adult stage. Our data suggest that in addition to selected target-site resistance mutations, agricultural pollutants may also favor cuticle, metabolic and synaptic transmission-based resistance mechanisms. These results emphasize the need for integrated resistance management strategies taking into account agriculture activities.

**Electronic supplementary material:**

The online version of this article (doi:10.1186/s13071-014-0480-z) contains supplementary material, which is available to authorized users.

## Background

Malaria vector control programmes have shown success in the last few years through the use of chemical insecticides presented as insecticide treated bed nets (ITNs) or indoor residual spraying (IRS). These tools have been shown to be effective in reducing malaria transmission [1,2]. However, this success is now threatened by the rise of resistance in malaria vectors across Africa [3]. Over the years, resistance of malaria vectors to insecticides has been attributed to the intensive use of ITNs and IRS [4-7]. However, an increasing number of studies suggested that the use of pesticides in agriculture contributes to the selection of resistance in mosquitoes, threatening the efficacy of vector control interventions [8-14]. In Africa, agriculture sector accounts for 60% of employment with large areas of intensive agriculture. In addition to intensive agriculture, the rapid growth of African cities also led to the development of small-scale urban agriculture with uncontrolled use of pesticides [14,15]. As most insecticides used in agriculture are of the same chemical classes and share the same targets and modes of action as those used for vector control, they have the potential to select for resistance in mosquitoes [12]. Indeed, adults resting on crops treated with pesticides may undergo a significant selection pressure. However, the agricultural selection pressure may differ from the ones caused by vector control activities in term of mode of exposure (larvae likely exposed to agrochemical leachates versus adults only targeted by vector control) and the nature of chemicals (complex mixtures of agrochemicals versus single insecticide molecules), which may select for different resistance phenotypes although this has not been deeply investigated.

Resistance of mosquitoes to chemical insecticides involves various mechanisms. The best-known mechanism is target-site mediated resistance which involves non-synonymous mutations affecting the proteins targeted by insecticides. Several target-site mutations have been described in malaria vectors including the *kdr* (knock down resistance) mutations of the sodium channel conferring resistance to pyrethroids and DDT [16-19], the *ace1* mutation of the acetylcholinesterase conferring resistance to organophosphates and carbamates [10,20,21] and the *rdl* mutation of the ɤ-aminobutyric acid (GABA) receptor conferring resistance to cyclodiens [22]. Although more complex and less well characterized at the molecular level, metabolic resistance is also frequent in mosquitoes and consists of an improved bio-degradation of the insecticide through metabolic processes. This mechanism classically involves ‘detoxification’ enzymes such as cytochrome P450 monooxygenases (P450s or *CYPs* for genes), glutathione S-transferases (GSTs) and carboxy/cholinesterases (CCEs) [12,23,24], although other enzyme families may also be involved. To date several candidate genes encoding detoxification enzymes have been identified in resistant malaria vectors and some have been functionally validated as insecticide metabolizers [25-29]. Other resistance mechanisms such as cuticle alteration, altered transport and sequestration are also likely to occur in resistant mosquito populations but their molecular characterization remains limited.

In Africa, resistance to insecticides such as DDT and pyrethroids has frequently been associated with pesticide usage in agriculture [11,14,30,31]. In Benin *An. gambiae* females were reported to frequently lay their eggs in breeding sites located around agricultural settings suggesting that larvae may undergo a selection pressure from agricultural pesticides, favouring the emergence of resistance [8]. In Tanzania, the resistance of *Anopheles arabiensis* to pyrethroids through metabolic resistance was associated with pesticide usage in an intensive agriculture area [30]. More recently, another Tanzanian field study comparing urban, agricultural and low pollution areas pinpointed the elevated resistance level of *An. gambiae* found in proximity of intensive agriculture and identified candidate genes associated with the use of pesticides in agriculture and insecticide resistance [32].

The use of agrochemicals may affect the resistance of mosquitoes to vector control insecticides in multiple ways review in [12]. First, insecticides used to protect crops and having the same targets and mode of action of those used for vector control may directly select for resistance mechanisms in mosquitoes. In addition, insecticides specifically used in agriculture may also select for cross-resistance mechanisms to those used for vector control. Finally, several studies have demonstrated that mosquito larvae exposed to sub-lethal doses of pollutants, herbicides or pesticides frequently display a higher tolerance to insecticides through the induction of their detoxifying system and possibly other mechanisms [33-35]. Recent data revealed that *An. gambiae* larvae exposed to a sub-lethal mixture of agrochemicals during their development show a strong increase of tolerance to deltamethrin at the larval stage and that this transient effect is passed through the adult stage (I. Akhouayri, personal communication). Although transient, such phenotypic plasticity may modulate the selection of particular resistance mechanisms by insecticides. Indeed, a study combining exposure to a non-toxic pollutant and selection with permethrin across several generations revealed different gene expression profiles between mosquitoes exposed to the pollutant before permethrin selection and those solely selected with permethrin [36]. Although the situation *in natura* is more complex, with chemical mixtures varying through time and space, the presence of agrochemicals in aquatic mosquito breeding sites is likely to affect the selection of genes conferring resistance of adult mosquitoes to insecticides used for vector control.

In this context, the present study aimed at investigating the potency of agrochemicals in contact with *An. gambiae* larvae to select for resistance mechanisms in adults to the chemical insecticides used in vector control. For achieving this, an urban population of *An. gambiae* not previously exposed to agrochemicals and displaying low resistance levels to insecticides was artificially selected at the larval stage with a mixture of agrochemicals through several generations. Constitutive resistance levels of adults to three insecticides (deltamethrin, DDT and bendiocarb) were compared between the selected and the non-selected strain across the selection process and resistance mechanisms were investigated at the molecular level. Results are discussed with regards to the contribution of agriculture in the emergence and spread of insecticide resistance mechanisms in malaria vectors.

## Methods

### Mosquitoes and selection procedure

An *An. gambiae* population collected in summer 2011 from an urban site of Dar es Salaam (Ilala district, GPS coordinates S6.84643, E39.18285 as described in [32]) in east Tanzania was stabilized in the laboratory for 3 generations and then used as a parental strain for the selection experiment (generation G_0_). To our knowledge, this population was not subjected to any recurrent agrochemical pollution. Mosquito control in this area consisted in IRS and ITN at a high coverage and malaria prevalence is around 3% [32]. A recent study indicates that this population displays a low resistance level to pyrethroid and DDT at the adult stage [32]. This population was composed of a mixture of *An. gambiae sensu stricto* (*s.s.*) and *An. arabiensis*, the former carrying the L1014S ‘*kdr east’* mutation at a low frequency (1.6%, [32]). No *ace1* mutation was detected in this population [32]. Only *An. gambiae s.s.* were used to constitute the parental strain used for selection experiments. During the selection process, mosquitoes were reared in standard insectary conditions as described in [32]. Selection procedure consisted in exposing early L3 larvae for 24 h to a mixture of chemicals commonly used in agriculture at each generation. Agrochemical mixture was composed of various insecticides from different classes (organochlorines, organophosphates and pyrethroids), common pyrethroid metabolites [25] together with two herbicides (glyphosate and atrazine). These chemicals were chosen according to data gathered from local pesticide shops and individual farmers during a survey conducted in 2011 in northeast Tanzania (Theresia Nkya, unpublished data). The proportion of the different chemicals was determined according to concentrations classically found in water analyses in intensive agriculture areas and the relative toxicity of each compound. Composition of the agrochemical mixture is described in Table 1. For selection, the stock solution was diluted 10000 fold in order to reach a larval mortality between 50% and 80% after exposure. From the 7th generation, this concentration was raised by 1.5 fold in order to maintain the selection pressure above 50%. Selection was performed on replicates of 50 larvae in 50 mL. At each generation, a minimum of 1000 larvae were used for selection. Selection procedure was carried out for 20 successive generations. In parallel, the parental strain was maintained in similar conditions without selection pressure (population size ~500).

**Table 1:** **Composition of the pesticide mixture used for selection**

**Chemical class**	Mosquito larvae toxicity*	**Stock solution (μg/L)**	**1X dilution for selection (μg/L)**	**1.5X dilution for selection (μg/L)**
***Organochlorine insecticides***				
DDT	medium	2000	0.2	0.3
Endosulfan	medium	2000	0.2	0.3
Lindane	low-medium	10000	1	1.5
***Organophosphate insecticides***				
Chlorpyriphos	high	10	0.001	0.0015
Chlorfenvinphos	high	10	0.001	0.0015
***Pyrethroid insecticides***				
Deltamethrin	high	10	0.001	0.0015
Permethrin	medium-high	100	0.01	0.015
***Pyrethroid metabolites***				
3-phenoxybenzoic acid	not toxic	20000	2	3
3-phenoxybenzoic alcohol	not toxic	20000	2	3
***Herbicides***				
Glyphosate	not toxic	20000	2	3
Atrazine	not toxic	20000	2	3

* I. Akhouayri, unpublished data.

### Bioassays with insecticides

Comparative bioassays were conducted on the selected and the non-selected strain every 5 generations (G_0_ parental, G_5_, G_10_, G_15_, G_20_) to monitor the effect of the pesticide mixture on the resistance of adults to insecticides across the selection process. Three insecticides were used: the pyrethroid deltamethrin, the organochlorine DDT and the carbamate bendiocarb. In order to focus on constitutive resistance levels (inherited mechanisms), bioassays were performed on 3–5 days-old non-blood fed females not previously exposed to any xenobiotic (offspring of survivors for the selected strain). Bioassays were conducted according to WHO guidelines with plastic test tubes and insecticide impregnated papers at the following concentrations: 4% DDT, 0.05% deltamethrin, and 0.1% bendiocarb. Mosquitoes were exposed to each insecticide for varying durations (5, 10, 20, 30, 45 or 60 min). For each exposure time, 4 replicates of 25 mosquitoes were used. After exposure, mosquitoes were allowed to recover with a 10% glucose solution for 24 h before recording mortality. Mortality was corrected using Abbot’s Formula when the mortality rate of controls was between 5-20%. Every five generations, the lethal time to kill 50% of individuals (LT_50_) was calculated for each strain and each insecticide. The following bioassays could not be performed due to insufficient number of mosquitoes: deltamethrin G_5_, DDT G_10_ and bendiocarb G_10_.

### Species identification and kdr genotyping

Females from the initial parental strain (G_0_ individuals) and both the selected and the non-selected strains after 20 generations (G_21_ individuals) were subjected to species identification and *kdr* mutation detection. Genomic DNA samples from 30 individual mosquitoes from each strain were analysed. Genomic DNA was extracted on individual mosquitoes by grinding and heating the mosquito at 65°C for 30 minutes in 100 μl Bender buffer (0.1 NaCl, 0.2 M sucrose, 0.1 M Tris–HCl pH 7.5, 0.05 M EDTA pH 9.1, 0.5% SDS) according to the method described by [37]. Species identification was performed following the PCR-based method described by [38]. The detection of East *kdr* (L1014S) and West *kdr* (L1014F) mutations was performed using the TaqMan® PCR diagnostic assays described in [39] and a MX3005P qPCR system (Agilent technologies).

### Transcription profiling using microarrays

A microarray approach was used to identify adult transcription level variations associated with the selection of larvae with the agriculture pesticide mixture. The constitutive transcriptional profiles of the parental strain (G_0_ individuals) and the non-selected and selected strains after 20 generations (G_21_ individuals) were compared using the AGAM 15 K microarray (Agilent technologies, array design A-MEXP-2196) representing more than 15 K *An. gambiae* transcripts. For each strain, three biological replicates of total RNA were extracted from pools of 10 three day-old non-blood fed adult females using the RNAqueous-4PCR kit (Ambion). Total RNA was treated with DNaseI (Ambion) to remove genomic DNA. The quantity and integrity of RNA was analysed using a 2100 Bioanalyzer (Agilent). Hundred ng of total RNA were used for cDNA synthesis and T7-RNA amplification with Cy3- or Cy5-labelled CTPs using the ‘Two-color Low Input Quick Amp’ labelling kit (Agilent Technologies). Purified cRNAs were quantified and Cy3/Cy5 specific activity were measured using a Nanodrop ND1000 (NanoDrop Technologies). Pairwise hybridizations were performed between biological replicates of each strain with dye swaps for a total of 18 hybridizations (3 comparisons x 3 replicates x 2 dye swaps). Hybridizations, slide washes and scanning were performed according to manufacturer’s instructions. Spot finding, signal quantification and normalization were performed using the Agilent Feature Extraction software (Agilent Technologies). Normalized signal intensities were then loaded into Genespring GX version 12.5 (Agilent technologies) for further analysis. Spots showing a signal-to-noise ratio >2 for both colours were flagged ‘detected’ and only probes detected in at least 50% of hybridizations per comparison were considered for further analysis. For each pairwise comparison, transcription ratios (TRs) were log_2_ transformed and subjected to a one sample Student’s t-test against the baseline value of 0 (equal gene expression in both strains) with a Benjamini-Hochberg FDR multiple testing correction. Transcripts with a TR ≥3 fold in either direction and a t-test adjusted p-value ≤0.01 between the selected and both the parental and the non-selected strain were considered differentially transcribed following selection with the pesticide mixture. Differentially expressed transcripts were then assigned to distinct categories according to their putative function as follows: amino acid and protein metabolism; lipid and carbohydrate metabolism; cuticle; detoxification; immunity; nervous system/hormones/messengers; expression regulation/chaperonin; structure/housekeeping; other. Transcripts belonging to each category were then submitted to a clustering analysis based on the Euclidean distance between log_2_ TR across each condition using TM4 MeV (http://www.tm4.org/mev.html).

### Transcription profiling across the selection process using RT-qPCR

The transcription profile of candidate genes was further examined across the selection process by comparing their transcription level in the selected and the non-selected strains at generations 10, 15 and 21 by using RT-qPCR. RNA samples were extracted from three biological replicates of ten 3–5 days-old females per strain using the RNaqueous 4PCR total isolation kit (Ambion). Specific primers targeting each transcript were designed using NCBI Primer-Blast algorithm and their specificity checked against *An. gambiae* genome. Target transcripts included the two cytochrome P450s *CYP4J10* and *CYP6N1*, the sulfotransferase AGAP009553, the multicopper oxidase AGAP003738, the cuticle protein AGAP010123 and the two heat shock proteins AGAP004583 and AGAP012891. Reverse transcription and qPCR reactions were performed as described in [32]. Data analysis was performed according to the ΔΔ_Ct_ method taking into account PCR efficiency [40] and using the housekeeping genes encoding the ribosomal proteins L8 (AGAP005802) and S7 (AGAP010592) for normalisation. Three technical replicates were performed per biological replicate and results were expressed as mean transcription ratio ± SD relative to the non-selected strain.

## Results

### Impact of larval selection on adult insecticide resistance levels

**Figure 1:**
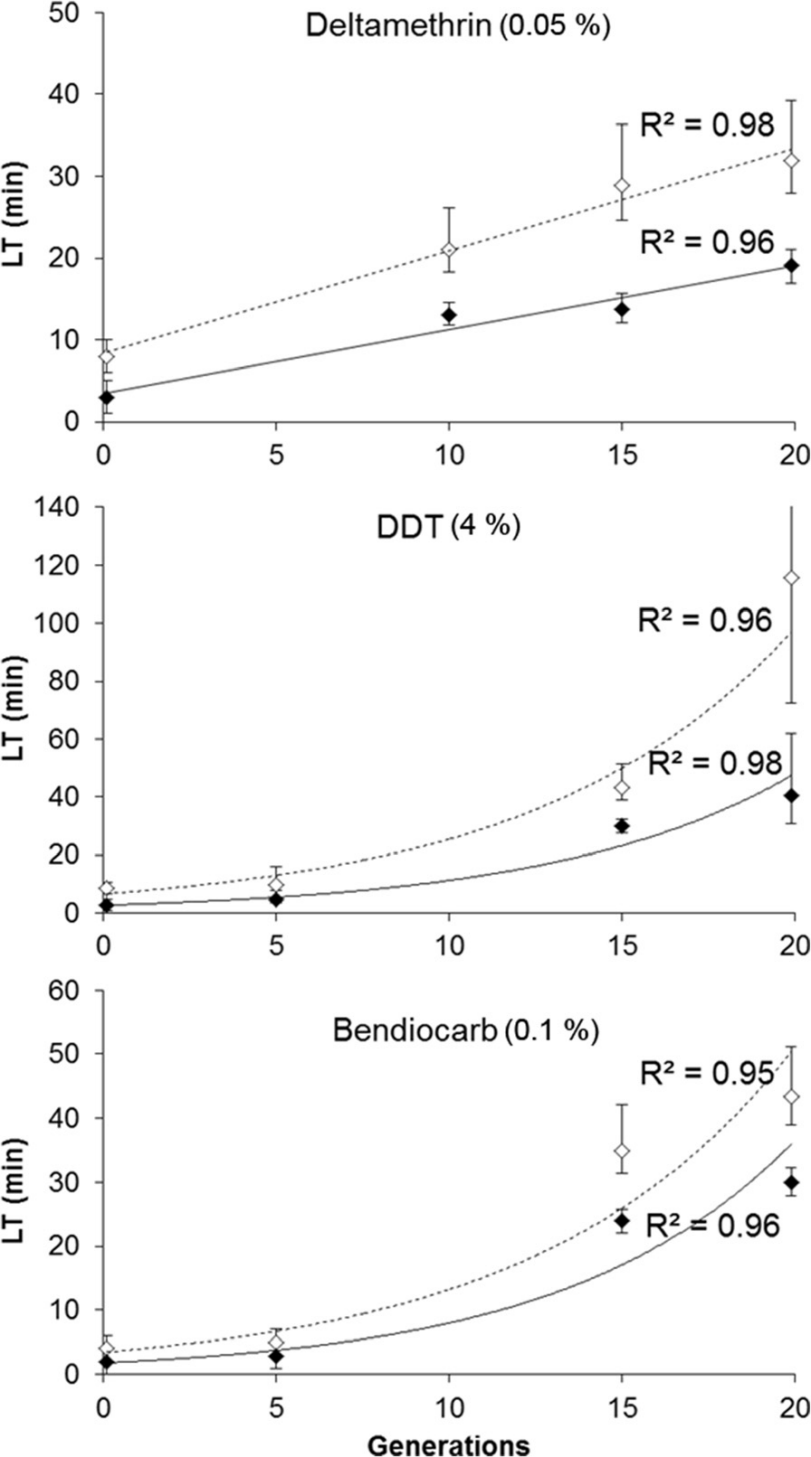
**Resistance levels of adults to three insecticides across the selection process.** Black lozenges represent the time necessary to kill 50% of individuals (LT_50_). White lozenges represent LT_90_. Error bars represent 95% confidence intervals. Generation 0 represents the parental strain. Solid and dotted lines represent the best fitting curves for LT_50_ and LT_90_ respectively.

Bioassays revealed that selecting *An. gambiae* larvae with the pesticide mixture increased their resistance to insecticides at the adult stage. A gradual increase of adult resistance to deltamethrin, DDT and bendiocarb was observed across the selection process (Figure 1). For deltamethrin, LT_50_ increased linearly from 3 min to 19.1 min (6.3 fold) after 20 generations of selection. A similar trend was observed when considering LT_90_ (4 fold). Resistance to DDT increased exponentially with LT_50_ rising from 2.8 min to 40.6 min after 20 generations (14.5 fold). A similar profile was observed with LT_90_ (13.6 fold). Likewise, an exponential increase of resistance to bendiocarb was observed with LT_50_ rising from 2 min to nearly 29.9 min (14.9 fold) and LT_90_ increasing by 10.8 fold.

### Species identification and kdr mutations frequency

PCR diagnostic assays confirmed that all individuals constituting the parental strain and the two derived strains were *An. gambiae sensu stricto*. The *kdr west* mutation (L1014F) was not detected in any strain. The frequency of the *kdr east* mutation (L1014S) was estimated at 1.6% in the parental strain and increased to 12.5% after 20 generations of larval selection with the pesticide mixture (Table 2). In contrast, the frequency of this *kdr* mutation decreased in absence of selection pressure as it was not detected in the non-selected strain after 20 generations (N = 30).

**Table 2:** **Evolution of kdr mutation frequency across generations**

**Strain**	**Genotype at position 1014**					**N**	**kdr allele frequencies (%)**		
	**LL**	**LS**	**SS**	**LF**	**FF**		**L (wildtype)**	**S (kdr east)**	**F (kdr west)**
**Parental**	29	1	0	0	0	30	98.4	1.6	0.0
**Non-selected**	30	0	0	0	0	30	100.0	0.0	0.0
**Selected G**_**20**_	30	3	3	0	0	36	87.5	12.5	0.0

### Transcriptome profiling after 20 generations of selection

**Figure 2:**
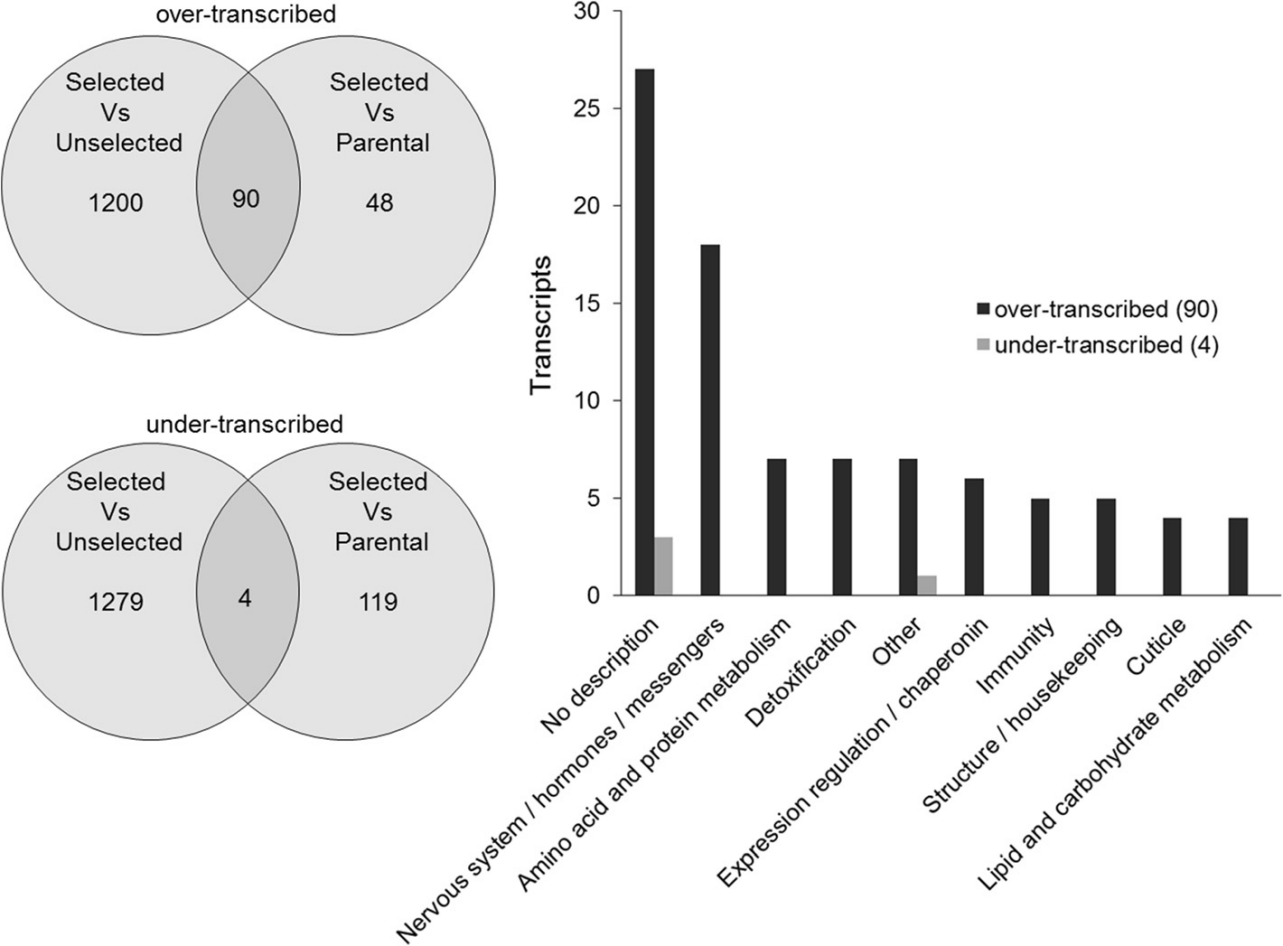
**Overview of transcripts differentially expressed in the selected strain.** The bar chart shows the biological categories represented by the 94 transcripts significantly over- or under-transcribed in the selected strain as compared to both the parental and the non-selected strains.

**Figure 3:**
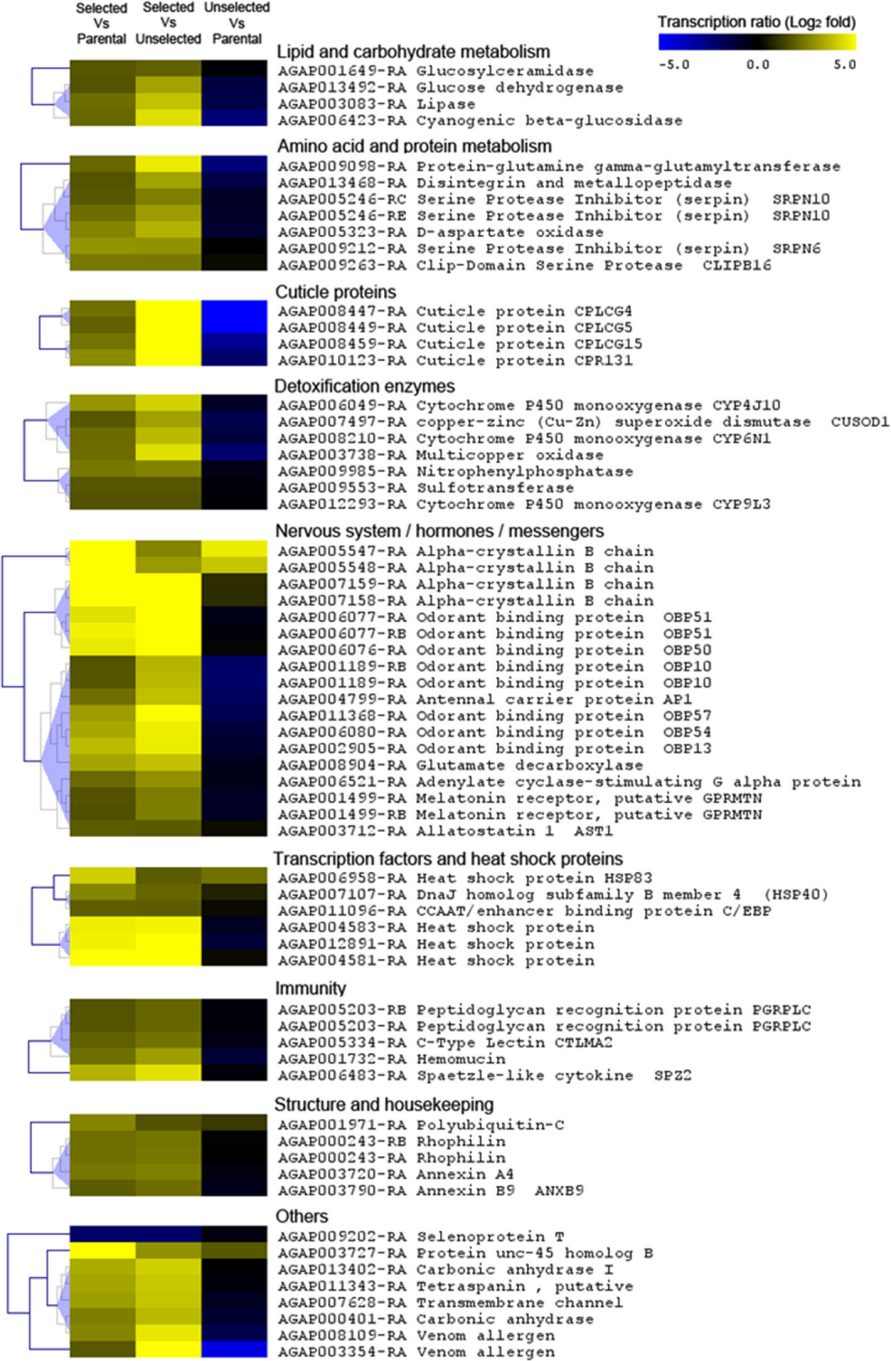
**Expression profile of all annotated transcripts differentially expressed in the selected strain.** Color scale indicates transcription levels (Log_2_ fold changes). The last column shows transcription level variations between the unselected strain and the parental strain. Transcripts were assigned to distinct biological functions based on their annotation (field ‘description’).

Microarray analysis detected 9088 transcripts showing consistent signal to noise ratio in all strains, including 2740 being differentially transcribed in the selected strain as compared to the parental or the non-selected strains (TR ≥3 and adjusted P value ≤0.01). Among those, 1338 transcripts were significantly over-transcribed in the selected strain versus other strains but only 90 transcripts were over-transcribed in the selected strain as compared to both the parental and the non-selected strains (Figure 2). Similarly, 1402 transcripts were under-transcribed in the selected strain versus other strains but only 4 of them were under-transcribed in the selected strain as compared to both the parental and the non-selected strains. Among the 94 transcripts differentially transcribed in the selected strain versus both the parental and the non-selected strains, only 64 were functionally annotated in Vectorbase. These transcripts encode protein families associated with various functions (Figure 2 and Additional file 1). Several of them were associated with nervous system functioning including multiple odorant binding proteins (18 transcripts). Others were associated with detoxification (7 transcripts), amino acid or protein metabolism (7 transcripts), lipid or carbohydrate metabolism (4 transcripts), expression regulation including heat shock proteins (6 transcripts), immunity (5 transcripts), cuticle (4 transcripts) and structure or housekeeping (5 transcripts). Most of these transcripts showed an over-transcription in the selected strain versus other strains but not in the non-selected strain as compared to the parental strain, suggesting that their increased expression is linked to the insecticide selection pressure (Figure 3). Among families classically associated with insecticide resistance, 3 cytochrome P450s (*CYP4J10*, *CYP6N1* and *CYP9L3*) were significantly over-transcribed together with other enzymes potentially involved in insecticide degradation pathways (1 multicopper oxidase, 1 sulfotransferase, 1 superoxide dismutase and 1 nitrophenylphosphatase). A strong over-transcription of 4 cuticle proteins (*CPLCG4*, *CPLCG5*, *CPLCG15* and *CPR31*) was also detected in the selected strain. An over-transcription of 3 transcripts encoding heat shock proteins showing high cDNA sequence identity among them was also detected. Among genes linked to nervous system functioning, 4 transcripts encoding alpha-crystallins were strongly over-transcribed in the selected strain with the two pairs AGAP007159/007158 and AGAP005547/005548 showing very high cDNA sequence similarity. Several transcripts encoding odorant binding receptors (*OBP51*, *OBP50*, *OBP10*, *OBP57*, *OBP54* and *OBP13*) and one antennal carrier protein (*AP1*) were also over-transcribed in the selected strain. Finally the only annotated transcript being strongly under-transcribed in the selected strain encoded a thioredoxin-like protein called selenoprotein T.

### Transcription profiling of candidate genes across the selection process

**Figure 4:**
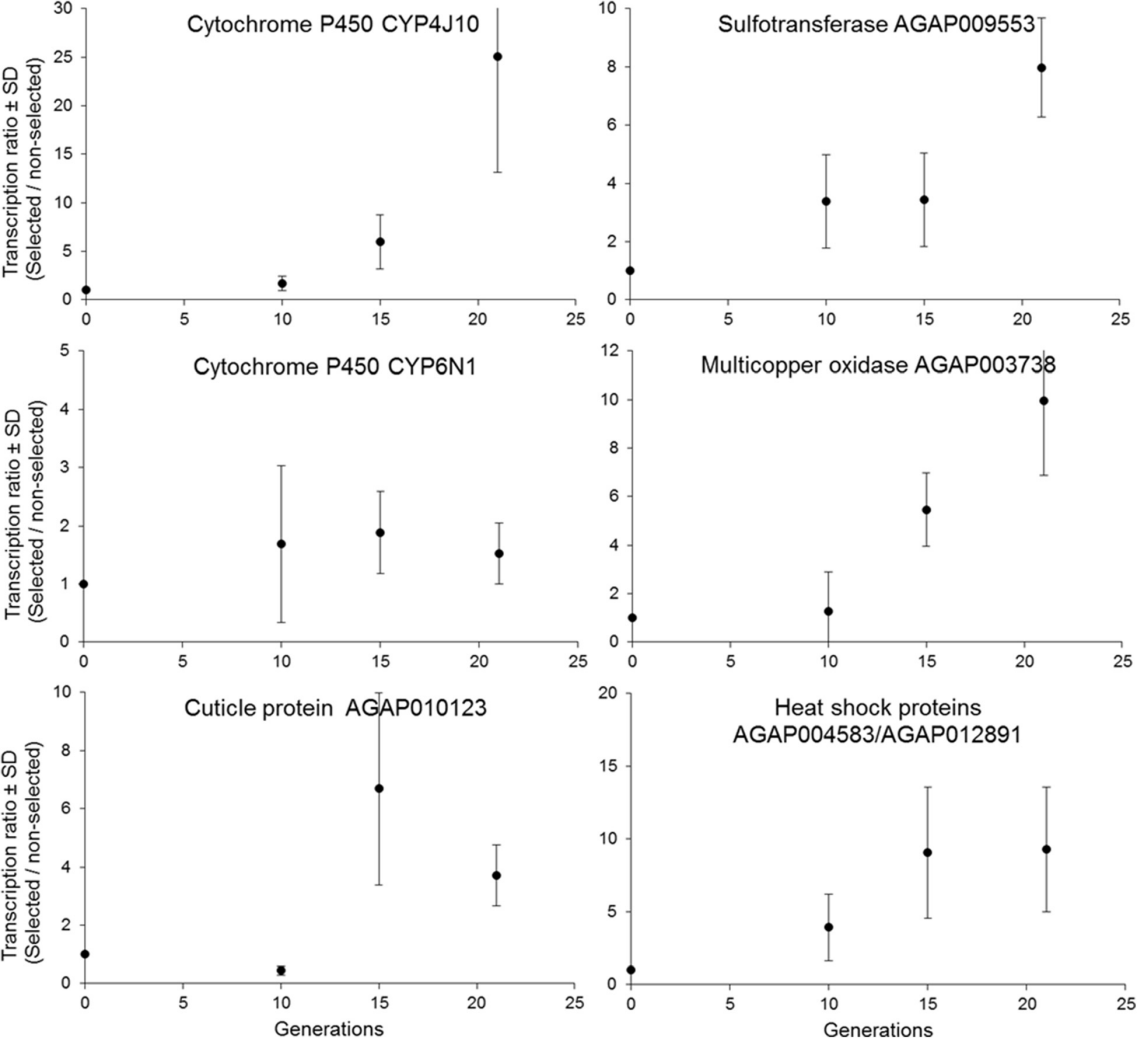
**Transcription profiles of candidate genes across the selection process.** Transcription levels were monitored by RT-qPCR at generations 10, 15 and 21 and are expressed as mean transcription ratio between the selected strain and the non-selected strain. The value of 1 at generation 0 represents the parental strain before selection.

Investigating the transcription profiles of six candidate genes across the selection process by RT-qPCR mostly confirmed microarray results (Figure 4). Among the six candidates the cytochrome P450 *CYP4J10*, the sulfotransferase AGAP009553, the multicopper oxidase AGAP003738 displayed a progressive increased expression through the selection process. Although primer design did not allow us to distinguish between the two heat shock proteins AGAP004583 and AGAP012891, their expression profile also showed a gradual increase across the selection process. Even if high stochastic variations affected the expression profile of the cuticle protein AGAP010123, its over-transcription was confirmed starting from generation 15. Conversely, the cytochrome P450 *CYP6N1* only showed a minor over-transcription in the selected strain with no gradual increase across the selection process.

## Discussion

It has frequently been suggested that pesticides and other chemicals used in agriculture can favour the evolution of resistance of mosquitoes to insecticides used for their control although only few studies have been dedicated to demonstrate this phenomenon [9,12,14]. One hypothesis states that mosquito larvae may be recurrently exposed to agrochemicals leaching from crop fields to their breeding sites leading to the selection of genes conferring resistance at the adult stage. The aim of the present study was to test this hypothesis in controlled conditions across multiple generations. To achieve this, a mixture of pesticides and agrochemicals classically found in water surrounding intensive agricultural areas was prepared. This mixture was then used for selecting a recently colonized *An. gambiae* population at the larval stage.

Bioassays confirmed that the repeated exposure of *An. gambiae* larvae to chemicals used in agriculture can select for resistance against vector control insecticides in adults. Monitoring resistance levels along the selection process revealed that resistance to multiple insecticide classes increased significantly after only a few generations of selection. Although some insecticides present in the selection mixture are identical or share the same targets as those used for adult bioassays (sodium channel for pyrethroids and DDT, and acetylcholinesterase for organophosphates and bendiocarb), such rapid and concomitant rise of resistance to all insecticides was not expected. This suggests that multiple resistance mechanisms expressed at the adult stage can be rapidly selected even when selection targets the larval stage. Such multi-resistant phenotypes can be the consequence of the selection of different mechanisms with limited trade-off between them or the selection of particular genes conferring cross-resistance to different insecticide classes. Although deltamethrin and DDT both target the voltage-dependant sodium channel, resistance developed more rapidly for DDT (linearly up to 6 fold for deltamethrin and exponentially up to 14 fold for DDT) suggesting the L1014S *kdr east* mutation is not the only cause of resistance. Regarding bendiocarb resistance, the absence of *ace1* mutation in the parental strain (N = 50, [32]) suggests that other resistance mechanisms have been selected. Finally, this multi-resistant phenotype may have also been favoured by the low dose of each insecticide in the mixture, not toxic if taken individually, but showing a strong synergistic effect in the pesticide mixture used for selection (Idir Akhouayri, unpublished data).

Transcriptomics identified various biological functions affected by selection with the pesticide mixture. As expected, multiple transcripts encoding enzymes classically involved in detoxification processes were over-transcribed in response to selection. The gradual increased expression level of the cytochrome P450 monooxygenase *CYP4J10* and the sulfotransferase AGAP009553 support their role in insecticide resistance as both have been associated with DDT resistance in *An. gambiae* [41]. The multicopper oxidase AGAP003738 is highly expressed in detoxification organs such as midgut and malpighian tubules [42] and has also been associated with DDT resistance [41]. In *Aedes aegypti*, a recent RNA-seq study identified a multicopper oxidase strongly over-transcribed in a strain resistant to the neonicotinoid insecticide imidacloprid [43] and slightly cross-resistant to DDT [44]. Surprisingly, no detoxification enzyme previously validated as deltamethrin or DDT metabolizer such as CYP6M2, CYP6P3 or GSTE2 [review in 26] were over-transcribed in the selected strain, suggesting that, besides the *kdr* mutation, agricultural selection pressure undertaken by larvae may select for genes different to those selected for by the use of insecticides for vector control.

In addition to detoxification genes, multiple cuticle proteins (*CPLCG4*, *CPLCG5*, *CPLCG15* and *CPR131*) increased in expression in the selected strain. The transcription level of two of them (*CPLCG4* and *CPLCG5*) strongly decreased in absence of selection, suggesting that these genes were already under selection in the initial parental strain. Indeed, cuticle plays a crucial role in protecting insects from their environment; hence changes in cuticle thickness or conformation have been suggested to contribute to resistance in mosquitoes [45-47]. For instance, *CPLCG4* has been frequently associated with insecticide resistance in malaria vectors [46,48]. A recent study supported its role in cuticle thickening, thus, possibly lowering the penetration of insecticides [49].

Two other functional categories were associated with pesticide mixture selection by transcriptomics. First, several transcription regulators, including five heat shock proteins (HSPs) and the CCAAT enhancer binding protein (C/EBP) AGAP011096 were over-transcribed after selection. These HSPs have been involved in response to thermal stress [50] and/or desiccation [51] but they were also found up-regulated in DDT-resistant field isolates [41] supporting their link with insecticide resistance and/or stress response. C/EBPs are known for their key role in gene expression regulation and the over-transcription of the only one C/EBP found in *An. gambiae* may reflect the need for mosquitoes under pesticide selection to over-express a broad panel of proteins.

Finally, several genes linked to nervous system functioning, perception and messenger signalling were over-transcribed in the selected strain. These included crystallins, which are known to play a key role in eye transparency and neuron functioning [52-54]. Also included were six odorant binding proteins (OBPs) with some of them previously found over-transcribed in an *An. gambiae* DDT-resistant populations originating from cultivated areas [41]. Others include one G protein receptor together with a G protein-stimulating adenylate cyclase, the neuropeptide hormone allatostatin and a glutamate decarboxylase which catalyses the biosynthesis of the neurotransmitter ɤ-aminobutyric acid (GABA) through glutamate decarboxylation. Such impact of agricultural pesticides on mosquito nervous system functioning was previously evidenced from field populations collected in intensive agriculture areas [32]. Although these data need to be functionally validated, these results suggest that, in addition to known target-site mutations, the differential expression of other genes encoding proteins involved in nervous system functioning may contribute to the resistance phenotype.

## Conclusion

Overall, our study demonstrated that mosquito larvae recurrently exposed to agricultural pesticide mixture can develop adult resistance mechanisms against vector control insecticides. This phenomenon occurred after only a few generations of selection and affected all tested insecticide classes. Transcriptomics revealed that a broad range of biological functions were affected including detoxification, cuticle, gene regulation and nervous system functioning. Although the present study did not allow dissecting the importance of each component of the mixture in the multi-resistant phenotype observed, the data confirmed the potential of agriculture in selecting for resistance in mosquitoes. These results strongly support the need for integrated vector resistance management strategies taking into account the use of pesticides in agriculture.

## Additional files

Additional file 1
**Table showing microarray data from all transcripts significantly differentially expressed in the selected strain.** For each transcript, biological category together with fold changes and P values are shown.

## Abbreviations

ITN: Insecticide-treated net
IRS: Indoor residual spraying
Kdr: Knock down resistance mutation
DDT: 4,4'-(2,2,2trichloroethane-1,1-diyl)bis(chlorobenzene)
GABA: Gamma-aminobutyric acid
P450: Cytochrome P450 monooxygenase enzymes
CYP: Genes encoding cytochrome P450 monooxygenase
CCE: Carboxy/choline esterase
GST: Glutathione S-transferase
ace1: Acetylcholinesterase resistance mutation
WHO: World Health Organization
LT_50_: Lethal time killing 50% of individuals
LT_90_: Lethal time killing 90% of individuals
cDNA: Complementary DNA
Cy3: Cynain-3 dye
Cy5: Cyanin-5 dye
cRNA: Complementary amplified RNA
TR: Transcription ratio
FDR: False discovery rate
RT-qPCR: Reverse transcription and quantitative polymerase chain reaction
NCBI: National Center for Biotechnology Information
OBP: Odorant binding receptor
HSP: Heat shock protein
C/EBP: CCAAT enhancer binding protein
